# Outbreak of Pneumococcal Meningitis, Paoua Subprefecture, Central African Republic, 2016–2017

**DOI:** 10.3201/eid2409.171058

**Published:** 2018-09

**Authors:** Matthew E. Coldiron, Oumar Touré, Thierry Frank, Nathalie Bouygues, Rebecca F. Grais

**Affiliations:** Epicentre, Paris, France (M.E. Coldiron, R.F. Grais);; Epicentre, Paoua, Central African Republic (O. Touré);; Institut Pasteur, Bangui, Central African Republic (T. Frank);; Médecins Sans Frontières, Paris (N. Bouygues)

**Keywords:** meningitis, pneumococcal meningitis, outbreak, meningitis/encephalitis, Streptococcus pneumoniae, bacteria, pneumococcal vaccines, Central African Republic

## Abstract

We report a pneumococcal meningitis outbreak in the Central African Republic (251 suspected cases; 60 confirmed by latex agglutination test) in 2016–2017. Case-fatality rates (10% for confirmed case-patients) were low. In areas where a recent pneumococcal conjugate vaccine campaign was conducted, a smaller proportion of cases was seen in youngest children.

In early January 2017, an abnormally large number of pneumococcal meningitis cases was reported at Paoua Subprefectural Hospital in northwestern Central African Republic. This region is at the southern edge of the traditional meningitis belt in Africa (*1*), and the hospital has been supported by the international medical humanitarian organization Médecins Sans Frontières since 2007. Routine data collected since 2012 showed a weekly maximum of 3 cases of pneumococcal meningitis (confirmed by latex agglutination test) and never >29 reported cases in any given 25-week period. A case-based meningitis surveillance system, including latex agglutination testing, was implemented in Paoua Subprefectural Hospital. All suspected cases in peripheral health centers were referred free of charge. We provide an epidemiologic description of this outbreak.

## The Study

The Central African Republic has experienced a series of crises over the past several decades. The most recent acute crisis began in 2013, when a series of armed rebellions led to multiple changes of power at the central level; a newly elected government took office in 2016, but many areas are still not secure. Thus, health systems, particularly in the rural periphery, are particularly weak.

Vaccination coverage remains low: nationwide administrative coverage for the first dose of 13-valent pneumococcal conjugate vaccine (PCV13) was 77% in 2016 and 52% for the third dose. PCV13 was introduced in the Paoua Subprefecture (population 236,000) in 2012. A series of multiantigen catch-up vaccination campaigns for children <5 years of age that included PCV13 was conducted by Médecins Sans Frontières in 2016. Several areas were inaccessible because of security concerns and were not included in the vaccination campaign.

Outbreaks of pneumococcal meningitis have been reported in Africa before and after introduction of pneumococcal vaccine. There was a recent large outbreak in Ghana (*2*) and several other smaller outbreaks in the traditional meningitis belt (*3*,*4*). Pneumococcal meningitis has case-fatality rates (CFRs) of 36%–66% depending on age, and the risk for sequelae is high (*5*). Outbreaks generally occur during the dry season (typical meningitis season), but these outbreaks are usually smaller than meningococcal outbreaks (*6*). Unlike meningococcal meningitis, there is no formal epidemic definition for pneumococcal meningitis, although a provisional definition was recently issued: a district or subdistrict with a weekly incidence of >5 suspected cases/100,000 population with >60% of confirmed meningitis cases caused by *Streptococcus pneumoniae* and >10 confirmed cases of pneumococcal meningitis (*7*,*8*).

During October 10, 2016–April 9, 2017 (epidemiologic week 41 in 2016 through epidemiologic week 14 in 2017), 251 suspected cases of meningitis were reported at Paoua Subprefectural Hospital: 200 cases from Paoua Subprefecture (attack rate 85 cases/100,000 population), 40 cases from outside Paoua Subprefecture, and 11 cases from villages that could not be identified (Figure). Lumbar puncture and latex agglutination testing were performed for 110 patients, of whom 101 had not received antimicrobial drugs before lumbar puncture.

**Figure:**
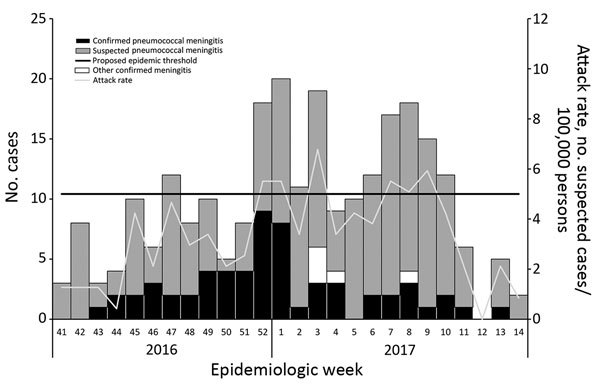
Cases of meningitis and weekly attack rate, Paoua Subprefecture, Central African Republic, 2016–2017.

Of 110 samples, 60 (55%) were positive for *S. pneumoniae* by latex agglutination test, 1 for *Neisseria meningitidis* strain NmW/Y, and 2 for *Haemophilus influenzae*. Two other samples showed a positive result, but the causative organism could not be identified. The remaining 45 samples were negative by latex agglutination test. Ten samples positive for *S. pneumoniae* by latex agglutination test were sent to the national reference laboratory (Institut Pasteur, Bangui, Central African Republic) where 6 were confirmed as serotype 1 *S. pneumoniae* by PCR, and 4 showed weak positive results for *S. pneumoniae* by PCR. Two samples negative by latex agglutination were also negative by PCR. *S. pneumoniae* represented 60 (92%) of 65 of all samples positive by latex agglutination during this period.

Overall, 9 patients died (CFR 3.6%). For case-patients with confirmed pneumococcal meningitis, 6 patients died (CFR 10.0%). For all case-patients, median length of treatment with ceftriaxone was 4 days (interquartile range 3–6 days). For case-patients with pneumococcal meningitis, median length of treatment was 10 days (interquartile range 9–12 days). Although information was incomplete, 25 case-patients with pneumococcal meningitis had documented evidence of treatment with dexamethasone.

Attack rates were highest for children <2 years of age (Table) when we considered all suspected cases and confirmed cases of pneumococcal meningitis. Areas targeted for the 2016 PCV13 vaccination campaign did not necessarily correspond to established political divisions. Thus, we were unable to calculate attack rates in vaccinated areas versus nonvaccinated areas because of lack of precise denominators. Nonetheless, in vaccinated areas, 5 (17%) of 30 confirmed cases were in children <5 years of age. In unvaccinated areas, 10 (36%) of 28 confirmed cases were in children <5 years of age. PCV13 vaccination status of case-patients was not recorded.

**Table:** Attack rates for pneumococcal meningitis, Paoua Subprefecture, Central African Republic, 2016–2017

Patient age, y	Overall cases of pneumococcal meningitis		Confirmed cases of pneumococcal meningitis	
	No. cases	Attack rate, no. cases/100,000 population	No. cases	Attack rate, no. cases/100,000 population
<2	61	301	12	59
2–4	22	82	4	15
5–14	38	62	24	39
15–29	79	124	11	17
30–44	37	96	7	18
>45	14	54	2	8

At a district level, this outbreak seems to have met the provisional definition of a pneumococcal meningitis outbreak, at least during epidemiologic weeks 52 in 2016 and weeks 1 and 3 in 2017, although it is unclear whether the criterion of >10 confirmed pneumococcal meningitis cases refers to a single week or overall during the outbreak. At a subdistrict level, only 2 subdistricts (Bah-Bessar, population 33,820; and Mia-Pendé, population 35,261) met the provisional definition at any point during the outbreak.

## Conclusions

We describe a pneumococcal meningitis outbreak in the Central African Republic in 2016–2017. This outbreak was not large, but it clearly was an abnormal event. Although pneumococcal outbreaks have been reported more frequently in recent years, outbreak definitions and guidance remain provisional and have been based on scanty data. We have highlighted a potential clarification that could be used in outbreak definitions in terms of the overall number of confirmed cases of pneumococcal meningitis.

We report low CFRs for case-patients with confirmed pneumococcal meningitis, which is in contrast to results of previous reports (*9*). These differences might have been caused by an extended duration of antimicrobial drug therapy and, at least for some case-patients, the addition of corticosteroids. Our small-scale observational data should not be overinterpreted, but length of therapy and utility of adjuvant corticosteroids were both highlighted as knowledge gaps in the provisional guidance document of the World Health Organization (*8*).

One limitation of our work was the level of biologic confirmation. However, the Pastorex Latex Agglutination Test Kit (Bio-Rad Laboratories, Marne-la-Coquette, France) we used has shown good performance in detecting *S. pneumoniae* (*10*). These kits were shipped and stored according to manufacturer’s instructions, and positive and negative controls were tested regularly according to standard procedures (*11*). We are reassured that at least a few samples underwent PCR confirmation and serotyping. Given that it appears that *S. pneumoniae* serotype 1 was the circulating serotype, differences in age distribution of case-patients seen between areas targeted and not targeted by the 2016 PCV13 catch-up vaccination campaign were likely caused by this intervention.

This outbreak highlights some of the difficulties inherent with performing surveillance in complex and insecure settings. The lack of infrastructure and laboratory capacity remain major obstacles to more precise characterizations of similar events. During this outbreak, it was not possible to perform cell counts or biochemical testing, which could have been useful. Increasing PCV13 coverage in the routine vaccination programs is the most efficient way to prevent future outbreaks, but given the overall context in the Central African Republic and other areas of the traditional meningitis belt, it would be prudent to consider formally evaluating (either by modeling or in real-life situations) the potential effects of reactive vaccination as an outbreak response.
